# Multivariate Pattern Classification of Facial Expressions Based on Large-Scale Functional Connectivity

**DOI:** 10.3389/fnhum.2018.00094

**Published:** 2018-03-19

**Authors:** Yin Liang, Baolin Liu, Xianglin Li, Peiyuan Wang

**Affiliations:** ^1^School of Computer Science and Technology, Tianjin Key Laboratory of Cognitive Computing and Application, Tianjin University, Tianjin, China; ^2^State Key Laboratory of Intelligent Technology and Systems, National Laboratory for Information Science and Technology, Tsinghua University, Beijing, China; ^3^Medical Imaging Research Institute, Binzhou Medical University, Yantai, China; ^4^Department of Radiology, Yantai Affiliated Hospital of Binzhou Medical University, Yantai, China

**Keywords:** facial expressions, fMRI, functional connectivity, multivariate pattern analysis, machine learning algorithm

## Abstract

It is an important question how human beings achieve efficient recognition of others’ facial expressions in cognitive neuroscience, and it has been identified that specific cortical regions show preferential activation to facial expressions in previous studies. However, the potential contributions of the connectivity patterns in the processing of facial expressions remained unclear. The present functional magnetic resonance imaging (fMRI) study explored whether facial expressions could be decoded from the functional connectivity (FC) patterns using multivariate pattern analysis combined with machine learning algorithms (fcMVPA). We employed a block design experiment and collected neural activities while participants viewed facial expressions of six basic emotions (anger, disgust, fear, joy, sadness, and surprise). Both static and dynamic expression stimuli were included in our study. A behavioral experiment after scanning confirmed the validity of the facial stimuli presented during the fMRI experiment with classification accuracies and emotional intensities. We obtained whole-brain FC patterns for each facial expression and found that both static and dynamic facial expressions could be successfully decoded from the FC patterns. Moreover, we identified the expression-discriminative networks for the static and dynamic facial expressions, which span beyond the conventional face-selective areas. Overall, these results reveal that large-scale FC patterns may also contain rich expression information to accurately decode facial expressions, suggesting a novel mechanism, which includes general interactions between distributed brain regions, and that contributes to the human facial expression recognition.

## Introduction

Facial expression is an important medium for social communication as it conveys information about others’ emotion. Humans can quickly and effortlessly decode emotion expressions from faces and perceive them in a categorical manner. The mechanism under which enables human brain achieving the efficient recognition of facial expressions is intensively studied.

The usual way in exploring facial expression perception is recoding the brain activity patterns while participants are presented with facial stimuli. Jin et al. (2012, 2014a,b) have made a lot of efforts on the stimulus presentation approaches with face stimuli. In order to accurately locate the increased neural activity in brain areas, functional magnetic resonance imaging (fMRI) technology is widely used. Using fMRI, earlier model for face perception is proposed by Haxby et al. (2000), in which they found a “core” and an “extended system” that participated in the processing of facial signals. Subsequently, the core face network, which contained the fusiform face area (FFA), the occipital face area (OFA) and the face-selective area in the posterior superior temporal sulcus (pSTS) have been widely discussed in facial expression perception studies and are considered as key regions (Haxby et al., 2000; Grill-Spector et al., 2004; Winston et al., 2004; Yovel and Kanwisher, 2004; Ishai et al., 2005; Rotshtein et al., 2005; Lee et al., 2010; Gobbini et al., 2011). Previous fMRI studies on facial expression perception mainly employed static expression images as stimuli (Gur et al., 2002; Murphy et al., 2003; Andrews and Ewbank, 2004). Because natural expressions include action, recent studies have suggested that dynamic stimuli are more ecologically valid than the static stimuli and the use of dynamic stimuli may be more appropriate to investigate the “authentic” mechanism of human facial expression recognition (Trautmann et al., 2009; Johnston et al., 2013). Recent studies with dynamic stimuli have found enhanced brain activation patterns compared with static stimuli and found that in addition to the conventional face-selective areas, motion-sensitive areas also significantly responded to facial expressions (Furl et al., 2012, 2013, 2015).

Most of the past fMRI studies on facial expression perception employed univariate statistics to analyze expression stimuli induced increments of neural activity in specific brain areas. Due to the expected existence of interactions between different brain areas, the analyses of functional connectivity (FC) attracted more and more attention, which is measured as the temporal correlations in the fMRI activity between distinct brain areas (Smith, 2012; Wang et al., 2016). Analysis of FC patterns has been applied in the recent studies of various objects categorization (He et al., 2013; Hutchison et al., 2014; Stevens et al., 2015; Wang et al., 2016), and it was generally observed that distinct brain regions are intrinsically interconnected. Considering these, FC patterns may also contribute to the facial expression recognition. A recent fMRI study on face perception employed FC patterns analysis to construct the hierarchical structure of the face-processing network (Zhen et al., 2013). However, it only focused on the FC patterns among the face-selective areas, the general FC interactions for facial expression recognition remained unclear. Consequently, exploring the whole-brain FC patterns during the processing of different expression information would be meaningful.

Machine learning techniques make use of the multivariate nature of the fMRI data and are being increasingly applied to decode cognitive processes (Pereira et al., 2009). Previous studies of facial expression decoding combined machine learning with multi-voxel activation patterns to examine the decoding performance in the specific brain areas. In these studies, Said et al. (2010) and Harry et al. (2013) respectively, highlighted the roles of STS and FFA in the facial expression decoding, and Wegrzyn et al. (2015) directly compared classification rates across the brain areas proposed by Haxby’s model (Haxby et al., 2000). Additionally, Furl et al. (2012) and Liang et al. (2017) showed that both face-selective and motion-sensitive areas contributed to the facial expression decoding. Considerable attention has been paid to activation-based facial expression decoding in individual brain areas; however, the potential mechanisms of expression information representation through the FC patterns remained unclear. Recently, a study by Wang et al. (2016) showed the successful decoding of various object categories based on the FC patterns. Their study motivated us to explore whether facial expression information can also be robust decoded from the FC patterns.

The present fMRI study explored the role of the FC patterns in the facial expression recognition. We hypothesized that expression information may also be represented in the FC patterns. To address this issue, we collected neural activities while participants viewed facial expressions of six basic emotions (anger, disgust, fear, joy, sadness, and surprise) in a block design experiment. Both static and dynamic expression stimuli were included in our experiment. After scanning, we conducted a behavioral experiment in accordance to previous study to assess the validity of the facial stimuli, in which we recorded the classification accuracy, the emotional intensity the participants perceived and the corresponding reaction times for each facial stimulus (Furl et al., 2013, 2015). A standard anatomical atlas [Harvard-Oxford atlas, FSL, Oxford University, Meng et al. (2014)] was employed to define the anatomical regions in the brain. We obtained the whole-brain FC patterns for each facial expression and then applied multivariate pattern analyses with machine learning algorithms (fcMVPA) to examine the decoding performance for facial expressions based on the FC patterns.

## Materials and Methods

### Participants

The data used in this study were collected in our previous study (Liang et al., 2017). Eighteen healthy, right-handed participants (nine females; range 20–24 years old) took part in our experiment. They were Chinese students who were recruited from the Binzhou Medical University. All participants were with no history of neurological or psychiatric disorders and had normal or corrected-to-normal vision. Participants signed informed consent before the experiment. This study was approved by the Institutional Review Board (IRB) of Tianjin Key Laboratory of Cognitive Computing and Application, Tianjin University.

### fMRI Data Acquisition

All the participants were scanned using a 3.0-T Siemens scanner with an eight-channel head coil in Yantai Affiliated Hospital of Binzhou Medical University. Foam pads and earplugs were used to reduce the head motion and scanner noise. Functional images were obtained using a gradient echo-planar imaging (EPI) sequence (TR = 2000 ms, TE = 30 ms, voxel size = 3.1 mm × 3.1 mm × 4.0 mm, matrix size = 64 × 64, slices = 33, slices thickness = 4 mm, slice gap = 0.6 mm). In addition, a three-dimensional magnetization-prepared rapid-acquisition gradient echo (3D MPRAGE) sequence (TR = 1900 ms, TE = 2.52 ms, TI = 1100 ms, voxel size = 1 mm × 1 mm × 1 mm, matrix size = 256 × 256) was used to acquire the T1-weighted anatomical images. The stimuli were displayed by high-resolution stereo 3D glasses within a VisualStim Digital MRI Compatible fMRI system (Choubey et al., 2009; Liang et al., 2017; Yang et al., 2018).

### Procedure

**FIGURE 1:**
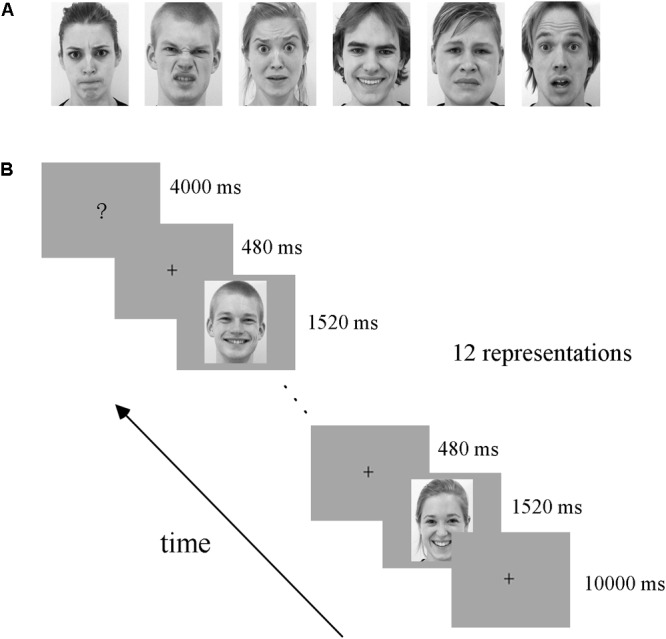
Exemplar facial stimuli **(A)** and schematic representation of the experimental paradigm **(B)**. All facial expression stimuli were taken from the ADFES database. The experiment employed a block design, including four runs. There was a fixation cross (10 s) before each block, and then 12 expression stimuli appeared. Subsequently, the participants completed a button task after each block to indicate their discrimination of the expression they had seen.

All facial expression stimuli were taken from the Amsterdam Dynamic Facial Expression Set (ADFES), which is a standard facial expression database containing both images and videos of basic emotions (van der Schalk et al., 2011). Video clips of 12 different identities (six males andsix females) displayed six basic emotions (anger, disgust, fear, joy, sadness, and surprise) were chosen. The exemplar stimuli for the six basic emotions are shown in **Figure 1A**. We created the dynamic expression stimuli by cropping all videos to 1520 ms to retain the transition from a neutral expression to the expression apex, and the apex expression image was used as the static stimuli (Furl et al., 2013, 2015).

The experiment employed a block design, with four runs. There were three conditions in our experiment: static facial expressions, dynamic facial expressions and dynamic expressions with obscured eye-region. Each condition included all six basic expressions: anger, disgust, fear, joy, sadness, and surprise. Data from the obscured condition were not analyzed in the current study but were included for the purpose of another study (Liang et al., 2017). In each run, there were 18 blocks (6 expressions × 3 conditions), each expression and condition appearing once. The 18 blocks were presented in a pseudo-random order to ensure that the same emotion or condition were not presented consecutively (Axelrod and Yovel, 2012; Furl et al., 2013, 2015). **Figure 1B** shows the schematic representation of the employed paradigm. At the beginning of each run, there was a 10 s fixation cross, which was followed by a 24 s stimulus block (the same condition and expression) and then a 4 s button task. Successive stimulus blocks were separated by the presentation of a fixation cross for 10 s. In each stimulus block, 12 expression stimuli were presented (each for 1520 ms) with an interstimulus interval (ISI) of 480 ms. During the course of each stimulus block, participants were instructed to carefully watch the facial stimuli, and after the block, a screen appeared with six emotion categories and corresponding button indexes to instruct the participants to press a button to indicate the facial expression they had seen in the previous block (Liang et al., 2017; Yang et al., 2018). Participants were provided with one response pad per hand with three buttons each in the fMRI experiment (Ihme et al., 2014), and they were pre-trained to familiarize the button pad before scanning. The total duration of the experiment was 45.6 min, with each run lasting 11.4 min. Stimulus presentation was performed using E-Prime 2.0 Professional (Psychology Software Tools, Pittsburgh, PA, United States).

After scanning, participants were required to complete a behavioral experiment outside the scanner in accordance to the previous studies (Furl et al., 2013, 2015). During it, we recorded their classification of emotion category, emotional intensity rating and the corresponding reaction times for each stimulus used in the fMRI experiment. The emotional intensity for each stimulus was rated on a 1–9 scale with 1 refers to the lowest and 9 refers to the highest emotional intensity (Furl et al., 2013). Each stimulus was presented once in a random order, with the same duration as in the fMRI experiment.

### Data Preprocessing

Functional image preprocessing was conducted using SPM8 software^1^. For each run, the first five volumes were discarded to allow for T1 equilibration effects. The remaining functional images were corrected for the slice-time and head motion. Next, the functional data were normalized by using the structural image unified segmentation. The high-resolution structural image was co-registered with the functional images and was subsequently segmented into gray matter, white matter and cerebrospinal fluid. And the spatial normalization parameters estimated during unified segmentation were applied to normalize the functional images into the standard Montreal Neurological Institute (MNI) space, with a re-sampled voxel size of 3 mm × 3 mm × 3 mm. Finally, the functional data were spatially smoothed with a 4-mm full-width at half-maximum Gaussian kernel.

### Construction of Whole-Brain FC Patterns

Estimation of the task-related whole-brain FC was carried out using the CONN toolbox^2^ (Whitfield-Gabrieli and Nieto-Castanon, 2012) in MATLAB. For each participant, the normalized anatomical volume and the preprocessed functional data were submitted to CONN. We employed the Harvard-Oxford atlas^3^ (FSL, Oxford University, Meng et al., 2014) as network nodes, which contained 112 cortical and subcortical regions. Time series of functional MRI signal were extracted from each voxel and averaged within each ROI for each condition. CONN implemented a component-based (CompCor) strategy to remove the non-neural sources of confounders. Principle components associated with white matter (WM) and cerebrospinal fluid (CSF) were regressed out along with the six head movement parameters, and the data were temporally filtered with band-pass filter 0.01 – 0.1 HZ as previously used for task-induced connectivity analysis (Wang et al., 2016). We conducted ROI-to-ROI analysis to assess pairwise correlations between the ROIs. For both static and dynamic facial expressions, we obtained six FC matrices (112 × 112) for each participant, one per emotion category. Second-level analysis was performed for each facial expression for the group comparisons of the differences in expression-related FC between ROIs (*p* < 0.001, FDR corrected for connection-level, two-sides).

### Across-Subject Expression Classification Based on the FC Patterns

**FIGURE 2:**
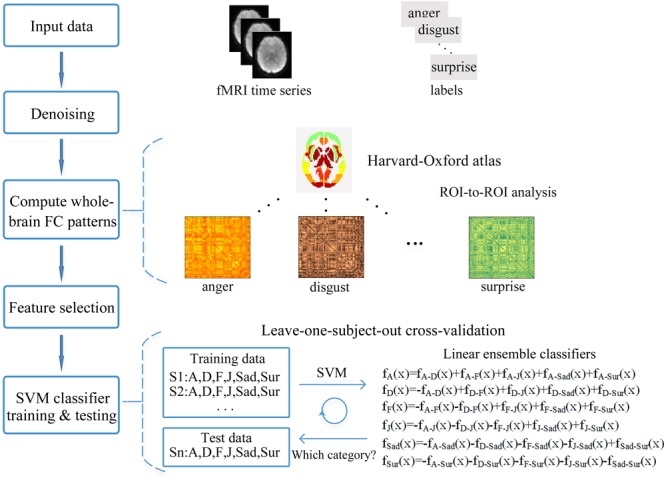
Framework overview of the fcMVPA. The pairs of preprocessed functional images and the corresponding labels of expression categories were used as the input data. Estimation of the FC patterns was performed using CONN toolbox. Denoising was used to remove unwanted motion, physiological and other artifactual effects from the BOLD signals before the connectivity measured. Then, the whole-brain FC patterns for each of the six facial expressions were computed using ROI-to-ROI analysis with the 112 nodes defined by the Harvard-Oxford atlas. Feature selection was performed with ANOVA only using the training data. Finally, the FC pattern classification of six facial expressions was carried out in a leave-one-subject-out cross-validation scheme with a SVM classifier.

We employed multivariate pattern analysis and machine learning to examine whether facial expression information could be decoded from the FC patterns (fcMVPA). **Figure 2** represents the framework overview of our fcMVPA classification. We performed a six-way expression classification separately for the static and dynamic facial expressions. Due to some evidence showed that the interpretation of the negative FCs remained controversial (Fox M.D. et al., 2009; Weissenbacher et al., 2009; Wang et al., 2016), we also focused mainly on the positive FCs in current study. For each category, we obtained 6216 [(112 × 111)/2] connections in total. We performed the procedure adopted in Wang et al. (2016) to obtain the positive FCs. For each category, we conducted a one-sample *t*-test across participants for each of the 6216 connections and retained FCs that had values significantly greater than zero. *P*-values were corrected for multiple comparisons with the false discovery rate (FDR) *q* = 0.01. This procedure identified 1540 positive FCs for anger, 1792 positive FCs for disgust, 1466 positive FCs for fear, 1838 positive FCs for joy, 1799 positive FCs for sadness and 1726 positive FCs for surprise for the static expressions, while for the dynamic expressions, it correspondingly identified 1944, 1798, 1696, 1703, 1608, and 1822 positive FCs for each of the six basic expressions. Pooling the positive FCs together separately for static and dynamic conditions, we obtained a total of 3014 (for static) and 2986 (for dynamic) FCs that were significantly positive for at least one expression (Wang et al., 2016). For classification, we employed a linear support vector machine (SVM) classifier as implemented in the LIBSVM^4^. A leave-one-subject-out cross-validation scheme (LOOCV) was used to evaluate the performance (Liu et al., 2015; Wang et al., 2016; Jang et al., 2017). For multi-class classification, this implementation used a one-against-one voting strategy. In each iteration of LOOCV, we trained the classifier in all but one participant and the remaining one was used as the testing set. During the classifier training, we first obtained 15 classifiers for each pair of expressions and then added these pairwise classifiers to yield the linear ensemble classifier for each expression. Feature selection was executed using ANOVA (*p* < 0.05), which was only performed on the training data of each LOOCV fold to avoid peeking. The statistical significance of the decoding performance was evaluated with permutation test, in which the same cross-validation procedure was carried out for 1000 random shuffles of class labels (Liu et al., 2015; Wang et al., 2016; Fernandes et al., 2017). The *p-*value for the decoding accuracy was calculated as the fraction of the number of accuracies from 1000 permutation tests that were equal to or larger than the accuracy obtained with the correct labels. If no more than 5% (*p* < 0.05) of the accuracies from all permutation tests exceeded the actual accuracy using correct labels, the results was thought to be significant.

## Results

### Behavioral Results

**FIGURE 3:**
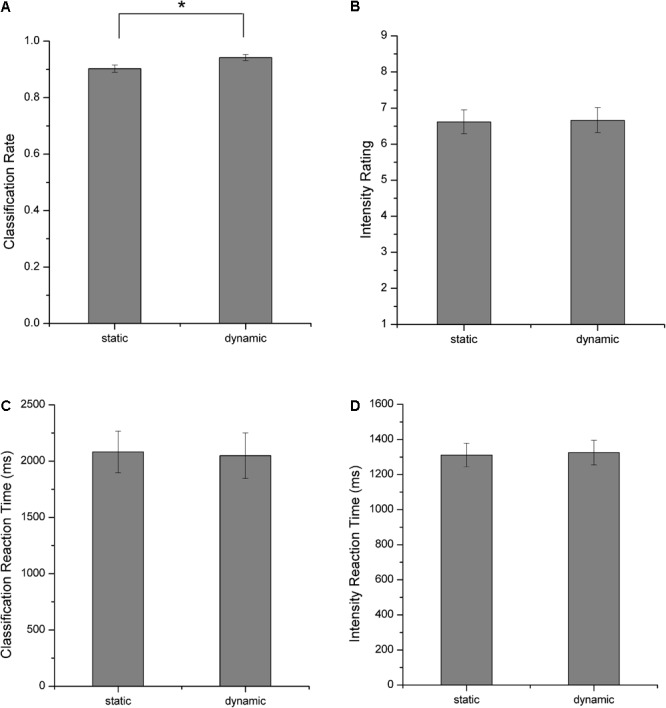
Behavioral results. **(A)** Classification rates, **(B)** perceived emotional intensities, **(C)** reaction times for facial expression classification, and **(D)** reaction times for emotional intensity rating. All error bars indicate the SEM. ^∗^Indicates statistical significance with paired *t*-test, *p* < 0.05.

Participants completed a behavioral experiment in which they classified the emotional categories and rated the perceived emotional intensities for each facial stimulus after scanning. **Figure 3** shows the behavioral results. These results verified the validity of the facial stimuli used in our experiment as both static and dynamic facial expression stimuli could be successfully classified with high accuracies (**Figure 3A**). A further comparison of the classification accuracies, intensity ratings and the corresponding reaction times between static and dynamic facial expressions, we found that participants showed higher classification accuracies for dynamic compared with static facial expressions [one-tailed paired *t*-test, *t*(17) = 3.265, *p* = 0.002]. For the emotional intensity and the reaction times, there were no significant differences.

### Whole-Brain FC Patterns for Each Facial Expression in Static and Dynamic Conditions

**FIGURE 4:**
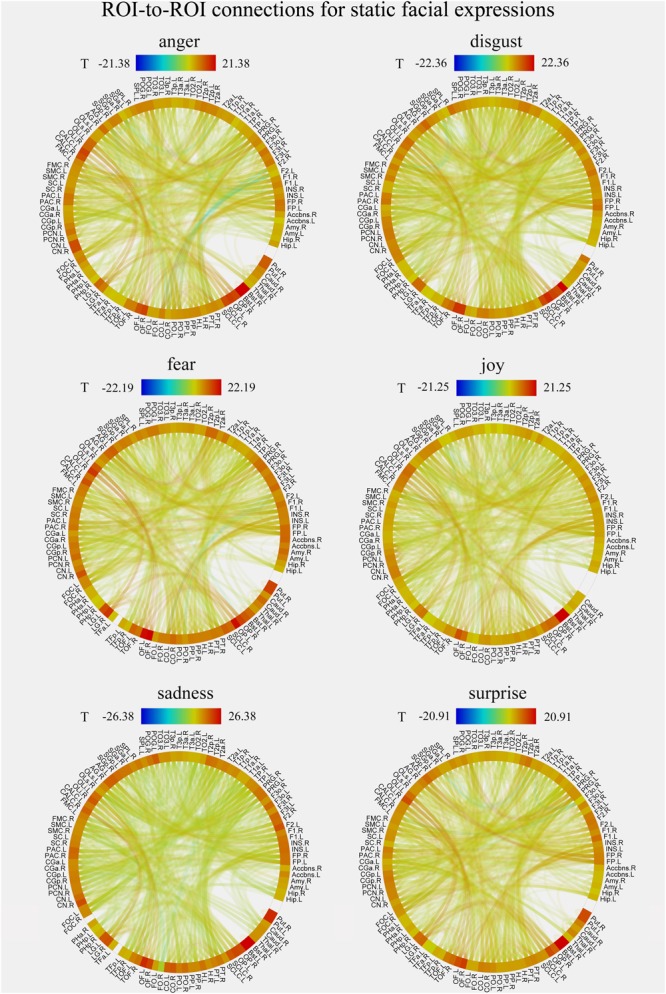
Group-level results of ROI-to-ROI connections for each facial expression (anger, disgust, fear, joy, sadness, and surprise) in static condition (*p* < 0.001, FDR corrected at the connection-level, two-sided). All ROIs are deriving from the Harvard-Oxford brain atlas and are labeled with the abbreviations for clarity.

**FIGURE 5:**
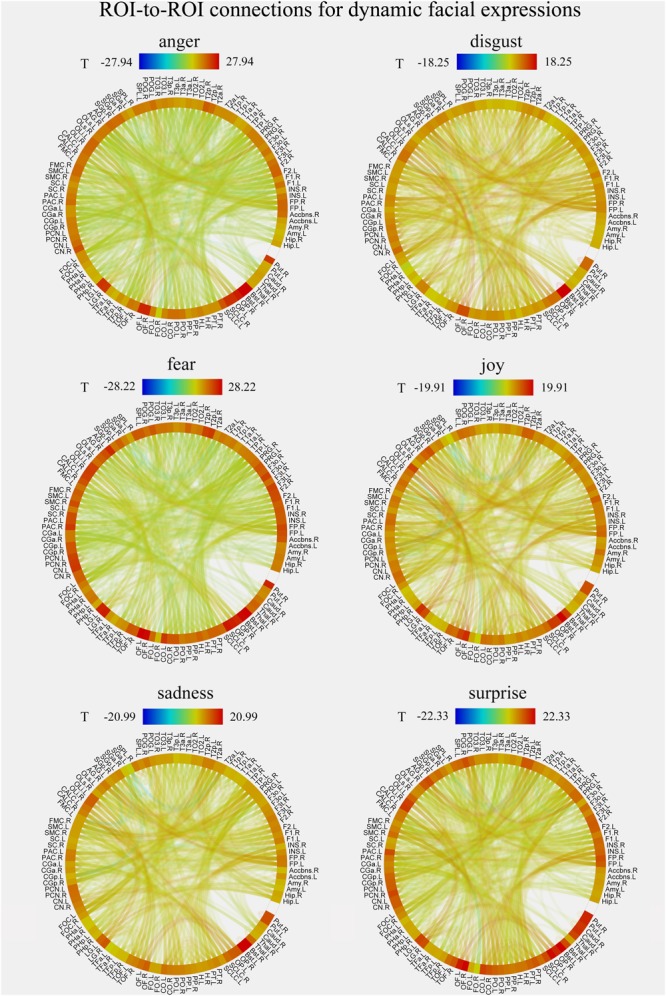
Group-level results of ROI-to-ROI connections for each facial expression (anger, disgust, fear, joy, sadness, and surprise) in dynamic condition (*p* < 0.001, FDR corrected at the connection-level, two-sided). All ROIs are deriving from the Harvard-Oxford brain atlas and are labeled with the abbreviations for clarity.

We constructed the whole-brain FC patterns for each facial expression separately for the static and dynamic stimuli. For each participant, we got 12 FC matrices with each contained 6216 [(112 × 111)/2] connections between the pre-defined 112 brain notes. Second-level analysis was performed for each facial expression for the group comparisons of the differences in these ROI-to-ROI functional connections. **Figures 4, 5** show the results of group-level analysis of the FC patterns for each facial expression (*p* < 0.001, FDR corrected for connection-level, two-sides).

### Facial Expression Decoding Based on fcMVPA

**FIGURE 6:**
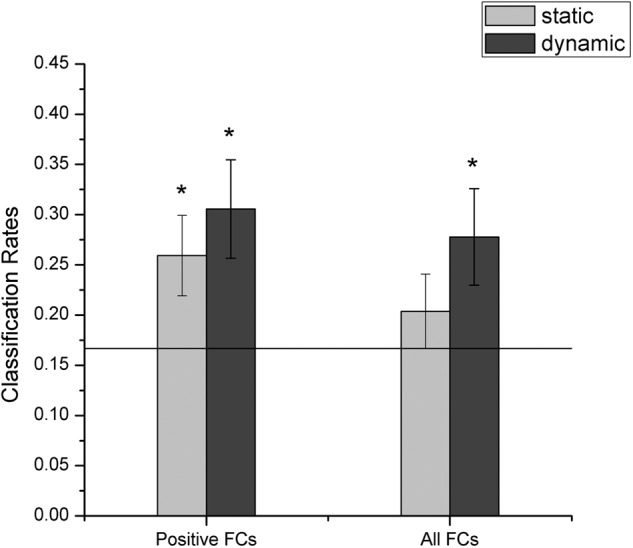
Accuracies of decoding static and dynamic facial expressions using fcMVPA. The black line indicates the chance level, and all error bars indicate the SEM. ^∗^Represents statistical significance over 1000 permutation tests.

In this section, we explored whether facial expressions could be decoded from the FC patterns using fcMVPA. Since the interpretation of the negative FCs remained controversial (Fox M.D. et al., 2009; Weissenbacher et al., 2009; Wang et al., 2016), we focused on the positive FCs in the fcMVPA classification. Separately for the static and dynamic conditions, we obtained the positive FCs for each facial expression using one-sample *t*-test across participants with multiple comparisons (FDR *q* = 0.01) and by pooling the positive FCs together, we obtained 3014 (for static) and 2986 (for dynamic) FCs for the classification of static and dynamic facial expressions (Wang et al., 2016). In the main results below, we used these positive FCs. For the multiclass facial expression classification, the performance was evaluated with the LOOCV strategy. As shown in **Figure 6** (left columns), we found that classification accuracies based on the FC patterns were significantly above the chance level for both static and dynamic facial expressions (*p* = 0.003 for static facial expressions and *p* < 0.001 for dynamic facial expressions, 1000 permutations), indicating that expression information could be successfully decoded from the FC patterns.

**FIGURE 7:**
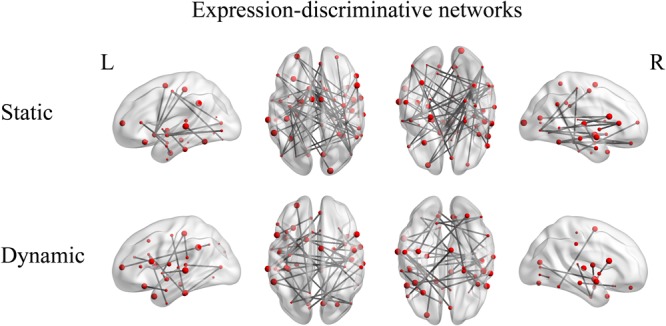
Expression-discriminative networks for the static and dynamic facial expressions. The coordinates of each node are according to the Harvard-Oxford brain atlas. The brain regions are scaled by the number of their connections and the results are mapped on the cortical surfaces using BrainNet Viewer.

Furthermore, we identified the expression-discriminative networks for the static and dynamic facial expressions, defined as FCs that contributed significantly in discriminating between different expression categories. Connections that were selected over all iterations of LOOCV feature selection (consensus features, ANOVA *p* < 0.05) composed the expression-discriminative networks. **Figure 7** shows the expression-discriminative networks for the static and dynamic facial expressions, all of which were widely distributed in both hemispheres. We summarized the brain regions that were involved in both static and dynamic expression-discriminative networks in **Table 1**. We found conventional face-selective areas, including the insula, inferior frontal gyrus, superior temporal gyrus, lateral occipital cortex (inferior occipital gyrus); temporal fusiform cortex (fusiform gyrus) and amygdala, which were commonly studied in previous fMRI studies on facial expression perception (Fox C.J. et al., 2009; Trautmann et al., 2009; Furl et al., 2013, 2015; Johnston et al., 2013; Harris et al., 2014). Moreover, we found the expression-discriminative networks contained brain regions far beyond these conventional face-selective areas. For instance, the middle temporal gyrus, which was reported sensitive to facial motion (Furl et al., 2012; Liang et al., 2017), was also included. Other regions that were not classically considered in previous fMRI studies on facial expression perception with activation measure were also included, such as the supramarginal gyrus, the lingual gyrus and the parahippocampal gyrus.

**Table 1:** Overlapping regions across static and dynamic expression-discriminative networks.

	Label	x	y	Z
L	Frontal pole	–25	53	8
L	Insular cortex	–36	1	0
R	Insular cortex	38	3	0
L	Inferior frontal gyrus, pars triangularis	–50	29	9
R	Inferior frontal gyrus, pars opercularis	52	15	16
L	Precentral gyrus	–34	–12	49
R	Temporal pole	41	13	–29
L	Superior temporal gyrus, anterior division	–56	–4	–8
R	Superior temporal gyrus, anterior division	57	–1	–10
L	Superior temporal gyrus, posterior division	–62	–29	4
L	Middle temporal gyrus, anterior division	–58	–4	–22
R	Middle temporal gyrus, posterior division	61	–22	–12
L	Inferior temporal gyrus, anterior division	–48	–5	–39
L	Inferior temporal gyrus, posterior division	–53	–28	–26
R	Inferior temporal gyrus, temporooccipital part	54	–50	–17
L	Postcentral gyrus	–39	–28	52
R	Postcentral gyrus	37	–27	53
L	Supramarginal gyrus, posterior division	–55	–46	34
R	Lateral occipital cortex, inferior division	45	–74	–2
L	Intracalcarine cortex	–10	–75	8
R	Intracalcarine cortex	12	–74	8
R	Frontal medial cortex	–5	44	–18
R	Juxtapositional lobule cortex (formerly supplementary motor cortex)	6	–3	58
R	Subcallosal cortex	6	20	–16
L	Frontal orbital cortex	–30	24	–16
R	Parahippocampal gyrus, anterior division	23	–8	–31
L	Lingual gyrus	–13	–66	–5
R	Lingual Gyrus	14	–63	–5
R	Temporal fusiform cortex, posterior division	–36	–24	–28
L	Planum polare	–47	–5	–8
R	Planum polare	48	–4	–7
R	Heschl’s gyrus (includes H1 and H2)	46	–17	7
L	Planum temporale	–53	–30	11
R	Planum temporale	55	–25	12
R	Supracalcarine cortex	9	–74	14
L	Hippocampus	–25	–23	–14
R	Amygdala	23	–4	–18

*The label and coordinates of each node are according to the Harvard-Oxford cortical and subcortical structural atlas*.

## Discussion

The main purpose of this study was to explore whether the FC patterns effectively contributed to human facial expression recognition. To address this issue, we employed a block design experiment and conducted fcMVPA. We obtained the whole-brain FC patterns for each facial expression separately for static and dynamic stimuli and found that both static and dynamic facial expressions could be successfully decoded from the FC patterns. We also identified the expression-discriminative networks for the static and dynamic facial expressions, composed of FCs that significantly contributed to the classification between different facial expressions.

### Facial Expressions Are Decoded From the FC Patterns

Using multivariate connectivity pattern analysis and machine learning algorithm, we found the successful decoding of both static and dynamic facial expressions based on the FC patterns.

Previous studies on facial expression recognition are dominated by identifying cortical regions showing preferential activation to facial expressions (Gur et al., 2002; Winston et al., 2004; Trautmann et al., 2009; Furl et al., 2013, 2015; Johnston et al., 2013; Harris et al., 2014). Although a few recent studies have started to explore the decoding of facial expressions, they only conducted activation-based classification analyses on individual brain regions (Said et al., 2010; Furl et al., 2012; Harry et al., 2013; Wegrzyn et al., 2015; Liang et al., 2017). The potential effects of the FC patterns on the facial expression decoding still undetected. Our study obtained the whole-brain FC patterns for each of the six basic expressions. Using fcMVPA, we found that expression information could be successfully decoded from the FC patterns. These results reveal that facial expression information may also be represented in the FC patterns, which add to the recently growing body of evidence for the large amount of information that the FC patterns contain for the decoding of individual brain maturity (Dosenbach et al., 2010), object categories (Wang et al., 2016), tasks (Cole et al., 2013) and mental states (Pantazatos et al., 2012; Shirer et al., 2012). Our study further provides new evidence for the potential of the FC patterns in the facial expression decoding. To summarize, our results suggest that the FC patterns may also contain rich expression information and effectively contribute to the recognition of facial expressions.

### Expression-Discriminative Networks Contain Brain Areas Far Beyond Conventional Face-Selective Areas

Neuroscience studies on facial expressions have paid considerable attention to the face-selective areas which exhibited selectivity to facial stimuli based on traditional activation analyses. Previous fMRI studies have indicated that face-selective areas are involved in the processing of facial expressions (Fox C.J. et al., 2009; Fox M.D. et al., 2009; Trautmann et al., 2009; Foley et al., 2011; Furl et al., 2013, 2015; Johnston et al., 2013; Harris et al., 2014). In our study, we obtained compatible results. We found the involvement of the face-selective areas in both static and dynamic expression-discriminative networks. In particular, the lateral occipital cortex (inferior occipital gyrus) for the early face perception (Rotshtein et al., 2005); the temporal fusiform cortex (fusiform gyrus) for the processing of facial features and identity (Fox M.D. et al., 2009) and the superior temporal gyrus for the processing of transient facial signals (Hoffman and Haxby, 2000; Harris et al., 2014) which together constitute the “core face network,” as well as a subset of brain areas in the extended face system including the amygdala, the insula and the inferior frontal gyrus that support the core system regions (Haxby et al., 2000; Fox C.J. et al., 2009; Trautmann et al., 2009; Johnston et al., 2013; Wegrzyn et al., 2015). Together, our results provide additional support for the importance of face-selective areas in the facial expression recognition with evidence from fcMVPA.

In addition, we found brain regions beyond these conventional face-selective areas participated in the expression-discriminative networks. The middle temporal gyrus, which was unanimously found sensitive to facial motion in the previous studies (Schultz and Pilz, 2009; Trautmann et al., 2009; Foley et al., 2011; Pitcher et al., 2011; Grosbras et al., 2012; Furl et al., 2013, 2015; Johnston et al., 2013; Schultz et al., 2013), was also included in our discriminative networks. Our results suggest the important role of the motion-sensitive areas in the processing of facial expressions. This is consistent with the previous evidence, which showed that motion-sensitive areas also represented expression information and contributed to the facial expression recognition (Furl et al., 2012; Liang et al., 2017). Moreover, other brain areas, which were found related to face or emotion perception in previous studies, were also included. For instance, the inferior temporal gyrus was related to emotional processing of faces in the study of effectivity connectivity on face perception (Fairhall and Ishai, 2007); the supramarginal gyrus and parahippocampal gyrus were found preference to face category by the fcMVPA (Wang et al., 2016); the lingual gyrus was reported in response to face stimuli, independent of emotional valence (Fusar-Poli et al., 2009) and the hippocampus was conventionally considered as an emotion-related region which was involved in emotion processing, learning and memory (Amunts et al., 2005; Xia et al., 2017). Furthermore, our study showed that brain regions, such as the postcentral gyrus and the Heschl’s gyrus, which were not classically considered in previous studies on facial expression perception with activation measure, were also included in the expression-discriminative networks. Together, these results suggest the potential effects of the activation-defined face-neutral regions in the recognition of facial expressions. To sum, our study showed the involvement of widespread brain regions beyond the conventional face-selective areas in the expression-discriminative networks, suggesting a potential mechanism which supports general interactive nature between distributed brain regions for the human facial expression recognition.

Moreover, it has been demonstrated a common neural substrate underlying the processing of static and dynamic facial expressions (Johnston et al., 2013). Our results support this idea with the analysis of FC, showing that a majority of common brain regions, which were involved in facial expression perception, are shared in the discriminative networks for both static and dynamic facial expressions.

In our present study, we employed comparable sample size as the previous fMRI studies on facial expression perception and MVPA-based analyses (Furl et al., 2012; Harry et al., 2013; Wegrzyn et al., 2015; Wang et al., 2016). Future studies with more samples may further improve the implementation of the classification scheme and boost the accuracy. Additionally, including of both Eastern and Western emotional expressions as stimuli in future studies could further investigate the potential cultural effect on facial expression recognition. In addition to the emotion information perceives from faces, body parts also convey emotion information (Kret et al., 2011, 2013). Therefore, further studies with comprehensive exploration of the FC patterns for both face and body emotions, investigating their similarities and differences may help to better understand human emotion perception.

## Conclusion

In summary, we show that expression information can be successfully decoded from the FC patterns and the expression-discriminative networks include brain regions far beyond the conventional face-selective areas identified in previous studies. Our results highlighted the important role of the FC patterns in the facial expression decoding, providing new evidence that the large-scale FC patterns may also contain rich expression information and effectively contribute to the facial expression recognition. Our study extends the traditional research on facial expression recognition and may further the understanding of the potential mechanisms under which human brain achieve quick and accurate recognition of facial expressions.

## Author Contributions

BL designed the study. YL, XL, and PW performed the experiments. YL analyzed the results and wrote the manuscript. YL and BL contributed to manuscript revision. All authors have approved the final manuscript.

## Conflict of Interest Statement

The authors declare that the research was conducted in the absence of any commercial or financial relationships that could be construed as a potential conflict of interest.

## Footnotes

1 http://www.fil.ion.ucl.ac.uk/spm/software/spm8/

2 http://www.nitrc.org/projects/conn

3 http://www.cma.mgh.harvard.edu/fsl_atlas.html

4 http://www.csie.ntu.edu.tw/cjlin/libsvm/
